# Impact Resistance and Energy Absorption Analysis of Crossover Frames with Different Materials

**DOI:** 10.3390/ma19091867

**Published:** 2026-05-01

**Authors:** Zhong-Hua Lv, Hao-Yu Wang, Guang-Ru Hua, Yan-Liang Yao, Min Xing, Zhi-Guo Zhang, Lei Wang, Yun-Xing Liu, Wei-Mu Jia, Lin-Yi Luo

**Affiliations:** 1 State Grid Liaoning Electric Power Company Limited Economic and Technology Research Institute, Shenyang 110027, China; 13840755187@163.com (Z.-H.L.); wanghaoyu@163.com (H.-Y.W.); 2 Department of Mechanical Engineering, North China Electric Power University, Baoding 071003, China; 13904148016@163.com (Y.-X.L.); mu980115@163.com (W.-M.J.); 18335447767@163.com (L.-Y.L.); 3 State Grid Liaoning Electric Power Company Limited Chaoyang Electric Power Supply Co., Chaoyang 122099, China; yaoyl_1990@163.com (Y.-L.Y.); xinmin1992@163.com (M.X.); 4 State Grid Liaoning Electric Power Supply Co., Ltd., Shenyang 110801, China; 15930511769@163.com (Z.-G.Z.); wangl_1988@163.com (L.W.)

**Keywords:** crossover frame, material comparison, energy absorption efficiency, numerical simulation

## Abstract

To address the insufficient impact resistance of crossover frames in UHV transmission projects, this study investigates the impact response and energy absorption of undamped frames using Q420 steel, 45 steel, 6061-T6 aluminum alloy, and EP fiberglass. An energy conversion model is established, and 1:20 scaled tests and finite element simulation are conducted over 0.5–5.0 m drop heights. The results show that Q420 steel achieves the highest energy absorption; aluminum alloy balances specific energy absorption and lightweight performance; 45 steel performs moderately; fiberglass reduces peak force but has limited energy dissipation and brittle fracture risk. The efficiency of metals rises with drop height, while that of fiberglass shows a slight decline. This work provides a theoretical basis for material selection and crossover frame optimization.

## 1. Introduction

With the continuous advancement of urbanization in our country, the scale of ultra-high-voltage (UHV) transmission projects has been expanding continuously, and transmission lines crossing railways, highways and other critical facilities during construction have become increasingly common. In such construction, the crossover frame serves as a temporary support system, and its stability and safety directly determine the safety of traffic operations below and personnel on site. Although crossover frames are temporary supporting structures used only during conductor deployment, their safety requirements are significantly stricter than those of general temporary structures. When transmission lines cross railways, highways, high-speed rails, and other critical facilities, accidental conductor breakage or runaway may generate intense impact loads. Existing conventional crossover frames are usually designed for static loads only, lacking effective impact resistance and energy dissipation mechanisms [1], which may lead to structural collapse, traffic paralysis, and serious safety hazards. Therefore, it is essential to study the impact response and energy absorption characteristics to improve the safety and reliability of temporary crossing structures under extreme dynamic loads.

In the research of crossover frame structures, Luo Yihua developed a self-elevating crossing frame incorporating self-supporting towers and beam arms, which improved the structural adjustability and adaptability. Wei S proposed a modular assembled crossing frame to address the complexity of erection, enhancing construction efficiency [2]. Meng Fanhao et al. designed a suspended crossing frame equipped with energy-absorbing devices to improve the dynamic response and energy dissipation performance under impact loads [3]. Existing studies have mainly focused on crossover frame structures and damping devices, while research on the impact resistance of crossing frames remains insufficient.

In terms of enhancing the impact resistance of structures, some researchers have attempted to introduce damping devices to improve energy dissipation capability [4,5]. Meng and others combined hydraulic energy-absorbing devices with suspended crossover frames, effectively enhancing the structure’s impact resistance [6]; Wieczorek and others explored the potential of using advanced materials in wheelchair frames to enhance vibration damping [7]. However, these studies mainly focus on the effects of dampers on structures and the inherent structure of crossover frames, and research on the energy absorption characteristics of crossover frames is still insufficient.

Regarding research on energy absorption effects, Xiao Y et al. adopted aluminum foam–polyurethane elastomer composites as buffer and energy-absorbing components, which realized satisfactory energy dissipation through deformation under impact loads [8]. Malewska E et al. used recycled polyurethane biofoam to prepare new energy-absorbing materials that can be applied to structural buffering and vibration reduction [9,10,11]. Florence A et al. designed a hybrid fiber honeycomb-core-reinforced polymer sandwich structure to improve the overall buffer and energy absorption performance by means of the honeycomb energy dissipation mechanism [12,13,14]. Karabanova L et al. prepared polyurethane-based nanocomposites used as noise and vibration damping energy-absorbing devices [15].

In the field of protective structures and buffer systems, numerous studies have confirmed that deformable energy-absorbing systems can effectively reduce impact loads and improve structural dynamic responses. Lee et al. verified through experiments and numerical simulations that deformable concrete barriers can dissipate impact energy via elastic–plastic deformation, significantly reducing peak impact force and improving structural safety [16]. Kim et al. analyzed structural failure and fragmentation under impact, further indicating that proper deformable energy-absorbing design can suppress brittle damage and reduce impact hazards [17]. These studies on deformable energy-absorbing systems provide important guidance for the impact-resistant design of transmission crossing frames, and demonstrate the necessity of introducing impact absorption mechanisms for temporary supporting structures.

At present, most existing studies related to crossover frames focus on structural performance analysis from a mechanical perspective, with insufficient attention paid to the mechanisms of impact buffering and energy dissipation. Systematic research on the energy absorption effects of the primary materials used in crossover frames remains relatively inadequate. When the conductor breaks suddenly, a large amount of kinetic energy is generated, and the tension in the cable also changes instantaneously. When this energy is transferred to the crossover frame structure, it will cause a huge impact on the frame. Therefore, to address the sudden impact condition of conductor breakage and prevent excessive deformation or even collapse of the structure due to instantaneous loads, it is necessary to introduce or design impact energy-absorbing structures and buffer devices in crossover frames as essential safety measures. This paper focuses on the energy absorption characteristics of crossover frames made of different materials, providing a basis for the optimal design of subsequent buffer-type crossover frames.

## 2. Basic Structure and Test Design of the Crossover Frame

### 2.1. Basic Structure and Parameters of the Crossover Frame

**Figure 1:**
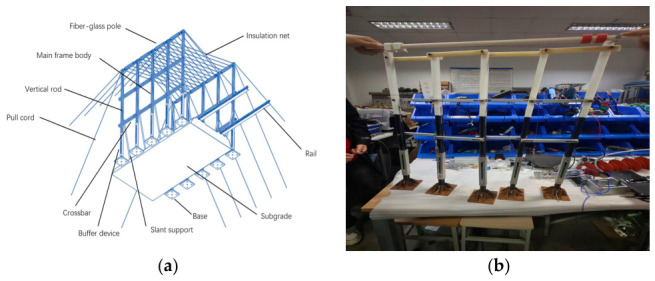
(**a**) Undamped crossover frame structure; (**b**) damped crossover frame prototype.

The main structure of the undamped crossover frame includes the support base, buffer device, main frame, and mesh covering. The structure of the undamped crossover frame is shown in Figure 1. The main parameters of the damping crossover frame are shown in Table 1. The view is shown from the lower left to the upper right.

According to the regulations of the “Electric Power Construction Safety Work Procedures” and the “Technical Guidelines for Tension Stringing Construction of Extra-High Voltage Overhead Transmission Lines”, the height of this crossover frame is designed to be 1000 mm, the width on one side is 680 mm, the spacing between vertical members is 170 mm, and the spacing of horizontal members is 250 mm.

Commonly used engineering materials at present include fiberglass (epoxy resin EP), aluminum alloy (6061-T6) [18,19], structural steel (Q420), and 45 steel. The parameters of each material are shown in Table 2.

### 2.2. Test Design of the Crossover Frame

Firstly, the research object and four experimental materials (Q420 structural steel, 45 steel, 6061-T6 aluminum alloy, and epoxy resin fiberglass) were identified. Subsequently, numerical simulations were adopted to determine the maximum drop height of the conductor and validate the structural safety within the experimental range. Due to the large span of the actual damped crossover frame, constructing a full-scale test environment requires substantial resources and high costs. Considering test efficiency and economic efficiency, a 1:20 scaled-down model is adopted for testing to ensure that performance indicators meet engineering requirements. The test adopts a controlled variable method, using crossover frames with the same structural configuration, only replacing the main body material as the single variable, to systematically compare the energy absorption performance of different materials and avoid structural damage caused by excessive tensile force during the test. A 1:20 scaled test model of the crossover frame was then established, and multi-height gradient drop impact tests were performed to acquire mechanical response and energy absorption data. Through multiple theoretical calculations and ANSYS (version 2022 R1, ANSYS, Inc., Canonsburg, PA, USA) simulation iterations, taking the Q420 structural steel crossover frame as the reference, the maximum safe drop height of the conductor impact is determined to be approximately 5 m. On this basis, the test drop height is set in the range of 0.5–5.0 m, with a step of 0.5 m for gradient loading. This setting can fully cover the stress characteristics under different impact energies, completely compare the energy absorption effects of different materials at different heights, and ensure that all test conditions are within the structural bearing safety range. Simulation verification shows that even at the maximum drop height of 5 m without additional damping, the crossover frame will not be pulled off, proving that the selected height interval does not exceed the ultimate bearing capacity of the structure.

The detailed test preparation and measurement procedures are as follows:

1. The scaled crossover frame is erected on a stable foundation.
2. Strain gauges(BX120-3AA, Hanzhong Jingcheng Electric Co., Ltd., Hanzhong, China) are attached to the nylon top crossbar of the crossover frame.
3. The strain gauges are connected to a dynamic signal testing and analyzing instrument(DH5922D, Donghua Testing Technology Co., Ltd., Jingjiang, China).
4. The instrument is further connected to a computer display terminal to form a complete data acquisition system.
5. During the test, the conductor is released from the preset height to fall freely and impact the crossover frame.
6. Transient stress and tension data are collected, displayed, and recorded in real time by the dynamic signal acquisition system.

**Figure 2:**
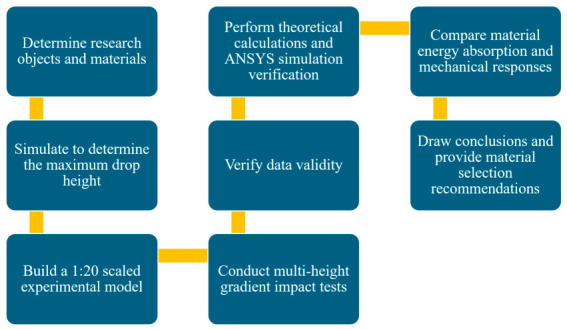
Experimental flow chart.

After verifying the validity of the experimental data, theoretical calculations and ANSYS simulations were carried out to analyze the energy absorption efficiency and mechanical characteristics of each material. Finally, the energy absorption performance and mechanical responses of different materials were compared, conclusions were drawn, and recommendations for material selection were proposed for undamped crossover frames. The detailed experimental flowchart is illustrated in Figure 2.

**Figure 3:**
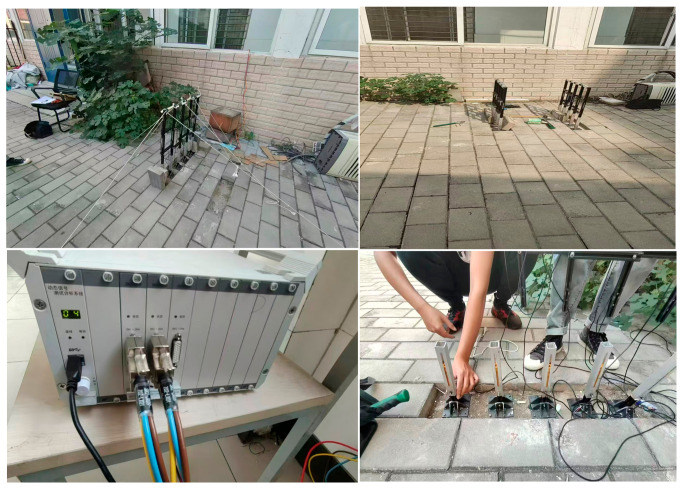
Actual photos of the experimental process.

The crossover frame Is erected on the ground. After standing the crossover frame, strain gauges are attached to the nylon rods and connected to a dynamic signal testing and analyzing instrument, which is further linked to a computer display terminal. The conductor is then released to fall and impact the crossover frame, and the computer display terminal analyzes the resulting stress conditions—these are the data obtained from the test. Figure 3 shows the actual photos of the experimental process.

This test and simulation verification are conducted to identify the damper with the optimal energy absorption performance that best matches the buffer-type crossover frame. The main structure of the buffer-type crossover frame includes a support base, a buffer device, a main frame body, and a mesh covering.

**Figure 4:**
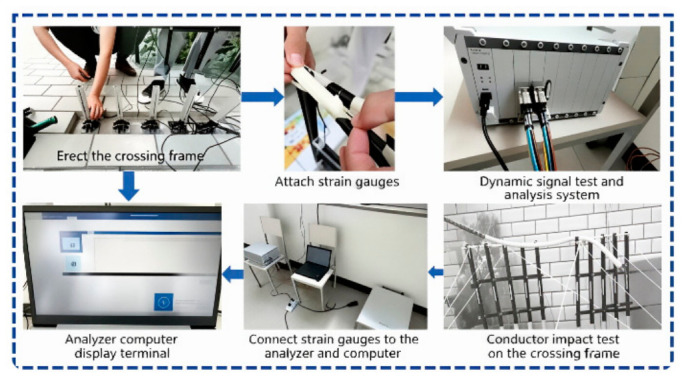
Test preparation.

Due to the large span of the damped crossover frame, constructing a full-scale test environment requires substantial resources and high costs. Considering test efficiency and economic efficiency, a 1:20 scaled-down model is adopted for testing to ensure that performance indicators meet the requirements. By setting different drop heights, the test investigates the crossbar tension under various damping devices, as well as the crossbar tension of each damping device under different damping coefficients.The test preparation setup is illustrated in Figure 4.

**Figure 5:**
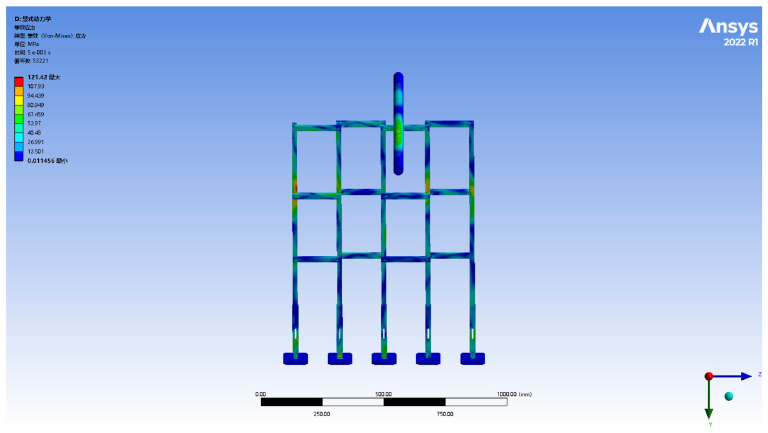
Determining the fall height in the crossbeam simulation. The Chinese text in the upper left corner of the figure represents the following meanings from top to bottom: D: explicit dynamic; equivalent stress; type: equivalent stress; units; time; cycle number; Max; Min.

To systematically compare the energy absorption performance of crossover frames made of different materials and prevent structural failure caused by excessive tensile force during testing, this study used crossover frames of the same configuration, only replacing the main material as the variable. Four commonly used engineering materials—fiberglass (epoxy resin EP), aluminum alloy (6061-T6), structural steel (Q420), and 45 steel—were selected to evaluate their impact resistance and energy absorption effectiveness. Through multiple theoretical calculations and simulation iterations, taking the structural steel Q420 crossover frame as the reference, the maximum drop height for the wire impact was determined to be approximately 5 m, with specific parameters shown in Figure 5. Based on this, the experiment set the drop height in the range of 0.5–5.0 m, incrementally increasing by 0.5 m. This scheme can comprehensively characterize the energy absorption characteristics of crossover frames made of different materials at various impact heights while also ensuring that all test conditions remain within the structural safety range. The simulation results indicate that the reference material (structural steel Q420) did not experience fracture under a 5 m drop impact. This demonstrates that within the selected drop height range of 0.5–5.0 m, the crossover frames made of these four materials all have sufficient strength to withstand conductor impact tension and do not exceed their ultimate load-bearing capacity.

### 2.3. Material Models and Experimental Data Acquisition

To systematically obtain the impact response and energy absorption characteristics of crossover frames made of different materials, 1:20 scaled drop-weight impact tests were conducted on four material configurations: Q420 structural steel, 45 steel, 6061-T6 aluminum alloy, and EP fiberglass. Tests were performed over a drop height range of 0.5 m to 5.0 m, with strain gauge measurements and dynamic signal acquisition used to record transient stress–time histories for each condition. These experimental data provide the basis for comparative mechanical analysis and subsequent finite element simulation validation.

Regarding constitutive modeling, appropriate simplifications were adopted in the numerical simulations according to the typical mechanical behavior of each material. Q420 structural steel and 45 steel were modeled using elastoplastic constitutive laws accounting for post-yield flow and hardening [20]. 6061-T6 aluminum alloy was represented by a rate-independent elastoplastic model reflecting its favorable ductility. EP fiberglass, as a brittle composite, was described by a linear elastic model with a defined failure threshold to capture its low stiffness and elastic-dominated energy absorption. The detailed mechanical response and energy absorption data derived from experiments for these materials are presented and discussed in Section 3.

### 2.4. Full-Scale Working Condition Verification and Dimensional Analysis

To clarify the extent to which the 1:20 scaled-model tests can inform full-scale engineering assessments, a rigorous dimensional analysis is first performed. The resulting scaling relationships are then used to interpret finite-element simulations of a 20 m full-scale crossover frame, thereby confirming the validity of the qualitative material comparisons drawn from the experiments.

For an impact event dominated by inertial and elastic forces, the Buckingham Π theorem dictates the scaling laws. Defining the length scale factor as $\lambda_{L}=L_{m}/L_{p}=1/20$, and keeping identical material density ($\lambda_{p}=1$) and normal gravity ($\lambda_{g}=1$), the required factors for strict elastic–inertial similarity are listed in Table 3 alongside the factors actually realized in the present experiment.

The drop height must remain identical at both scales ($\lambda_{h}\;=\;1$), ensuring $\lambda_{v}\;=\;1$. In the experiment, however, the drop height was reduced by the same geometric factor of 1/20, causing the impact velocity to be only $1/\sqrt{20}$ of the prototype value. This gravity-distortion effect fundamentally invalidates any direct quantitative extrapolation of force or energy readings. The following subsection provides an explicit quantification of the resulting force–scale mismatch.

#### 2.4.1. Quantitative Derivation of the Actual Force Scaling Factor

Using an equivalent elastic impact model, the peak impact force $F_{\max}$ can be expressed as follows:

(1) $$F_{max}=\sqrt{km}\cdot v$$

where k is the equivalent structural stiffness, *m* is the impact mass, and v is the impact velocity. The scaling factors for the components in Equation (1) are obtained from the experimental preset ratios $\lambda_{L}\;=\;1/20$, $\lambda_{E}\;=\;1$, and $\lambda_{h}\;=\;1/20$:

Cross-sectional area: $\lambda_{A}\;=\;\lambda_{L}^{2}\;=\;1/400$

Stiffness ($k\propto \frac{\mathrm{EA}}{L}$):

(2) $$\lambda_{k}=\frac{\lambda_{E}\lambda_{A}}{\lambda_{L}}=\frac{1\times 1/400}{1/20}=\frac{1}{20}$$

Mass ($m\propto \rho L^{2},\;\lambda_{\rho }=2$):

(3) $$\lambda_{m}=\lambda_{p}\lambda_{L}^{2}\approx \frac{1}{500}$$

Velocity ($v=\sqrt{2gh},\;r_{g}=1$):

(4) $$\lambda_{v}=\sqrt{\lambda_{g}\lambda_{h}}=\frac{1}{\sqrt{20}}$$

Substituting Equations (2)–(4) into Equation (1) gives the actual force scaling factor for this experiment:

(5) $$\lambda_{F}=\frac{1}{\lambda_{\sqrt{k}}}\cdot \lambda_{\sqrt{m}}\cdot \frac{1}{\lambda_{v}}=\sqrt{20}\cdot \frac{1}{\sqrt{500}}\cdot \sqrt{20}=\frac{2}{\sqrt{5}}\approx \frac{1}{1}$$

Equation (5) demonstrates that the peak force measured in the 1:20 scaled model is approximately 1/1 of the corresponding full-scale prototype value.

#### 2.4.2. Verification via Full-Scale Finite Element Simulation

**Figure 6:**
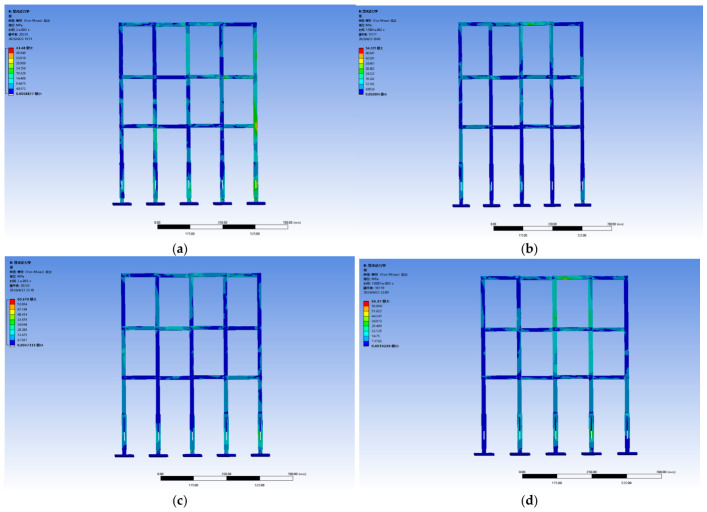
Stress condition under proportionally amplified overall load: (**a**) structural steel (**b**) aluminum alloy; (**c**) 45 steel; (**d**) fiberglass. The Chinese text in the upper left corner of the figure represents the following meanings from top to bottom: B: explicit dynamic; equivalent stress; type: equivalent stress; units; time; cycle number; Max; Min.

Despite the quantitative scaling mismatch, the scaled experiments still serve their primary purpose of relative material comparison, provided that the performance ranking obtained at the model scale is confirmed to hold at full scale. To verify this, a finite element model of a 20 m full-scale crossover frame was constructed, and impact simulations were carried out for the same four materials: Q420 structural steel, 45 steel, 6061-T6 aluminum alloy, and EP fiberglass. The resulting stress and deformation nephograms are shown in Figure 6.

The full-scale simulation results exhibit stress distribution patterns and deformation characteristics that are qualitatively consistent with those observed in the 1:20 scaled model. Importantly, the relative order of the four materials in terms of peak stress magnitude and energy absorption capacity Q420 steel > aluminum alloy > 45 steel > fiberglass is fully preserved between the two scales. This consistency confirms that the scaled tests capture the governing material-dependent mechanics and can validly guide material selection.

## 3. Impact Response Characteristics and Theoretical Analysis of Crossover Frames with Different Materials

### 3.1. Theoretical Calculation of Energy Absorption in Crossover Frame Crossbeams

In the study of impact energy absorption of the crossover frame, energy calculation is the core method for quantitatively evaluating the energy absorption performance of the system. By comparing the system’s initial total energy with the residual energy after impact (or directly measuring the dissipated energy), the energy absorbed by the crossover frame material can be obtained, and the energy absorption efficiency can be calculated. The wire–crossover frame–damper is considered a complete mechanical system, ignoring air resistance and energy loss at the support base.

#### 3.1.1. Energy Conversion During Impact Process

The conductor falls freely from a height h and hits the top crossbeam of the crossover frame. Ignoring air resistance, the gravitational potential energy of the conductor during the fall is converted into kinetic energy:

(6) $$E_{k}=mgh$$

where m is the mass of the wire and g is the acceleration due to gravity.

When the conductor contacts the crossarm, the crossarm undergoes elastic and subsequent elastoplastic deformation, and kinetic energy is converted into deformation energy of the crossarm.

To facilitate theoretical derivation, the crossarm is first assumed to exhibit linear elastic behavior with equivalent stiffness k; the elastoplastic stage will be further considered in the material constitutive description.

The deformation energy can be expressed as follows:

(7) $$U=\frac{1}{2}k\delta^{2}$$

where δ is the vertical displacement of the top crossbeam.

According to energy conservation, neglecting damping loss during impact:

(8) $$mgh=\frac{1}{2}k\delta_{max}^{2}$$

The maximum displacement can be obtained as follows:

(9) $$\delta_{max}=\sqrt{\frac{2mgh}{k}}$$

The maximum tension $T_{max}$ is:

(10) $$T_{max}=k\delta_{max}=\sqrt{2mghk}$$

The formula indicates that the maximum tension is proportional to $\sqrt{h}$ and $\sqrt{k}$.

The equivalent stiffness k depends on the geometry and elastic modulus E of the material (k∝E).

It should be emphasized that this elastic analysis provides a preliminary description; the actual structural behavior involves elastoplastic deformation after yielding, which will significantly affect the real stiffness and energy absorption characteristics.

#### 3.1.2. Characterization of Material Energy Absorption Capacity

The energy absorbed by materials under impact can be characterized by the area under the stress–strain curve [14,21].

For the elastic stage below the yield limit, the elastic strain energy density per unit volume is:

(11) $$\omega_{e}=\frac{\sigma_{y}^{2}}{2E}$$

where $\sigma_{y}$ is the yield strength and E is the elastic modulus.

When the stress exceeds the yield strength, the material enters the plastic stage, and plastic dissipation becomes the primary energy absorption mechanism.

The plastic dissipation energy per unit volume can be expressed as follows:

(12) $$\omega_{p}=\int_{\varepsilon_{y}}^{\varepsilon_{u}}\sigma \left(\varepsilon \right)d\varepsilon$$

where $\varepsilon_{y}$ is the yield strain, $\varepsilon_{u}$ is the ultimate strain, and σ(ε) is the flow stress.

The total energy absorbed by the structure is the product of the material volume V and the average strain energy density (elastic + plastic).

Under the same geometric conditions, the energy absorption capacity depends on the combined characteristics of strength, stiffness, and plastic deformability.

Materials with low elastic modulus tend to produce larger deformation, which helps reduce the peak impact force; materials with sufficient plastic deformability can achieve higher energy absorption through elastoplastic behavior.

### 3.2. Stress Response and Force Characteristics of Four Materials Under the Same Impact Load

**Figure 7:**
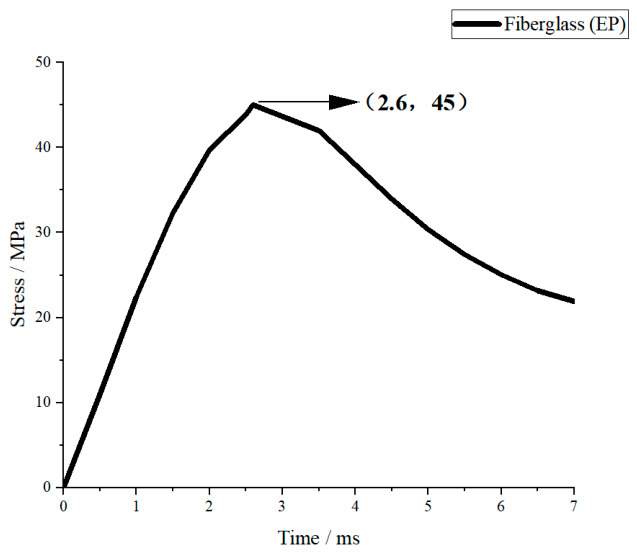
Fiberglass (EP) stress–time curve.

**Figure 8:**
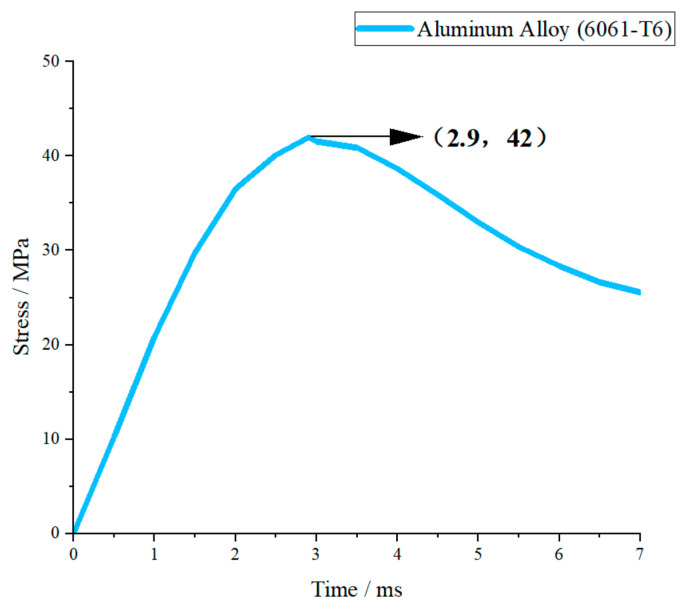
Aluminum alloy (6061-T6) stress–time curve.

**Figure 9:**
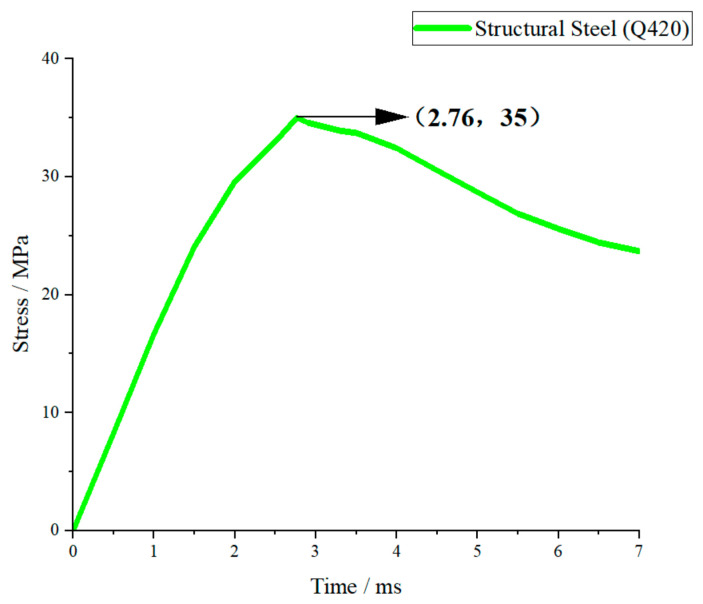
Structural steel (Q420) stress–time curve.

**Figure 10:**
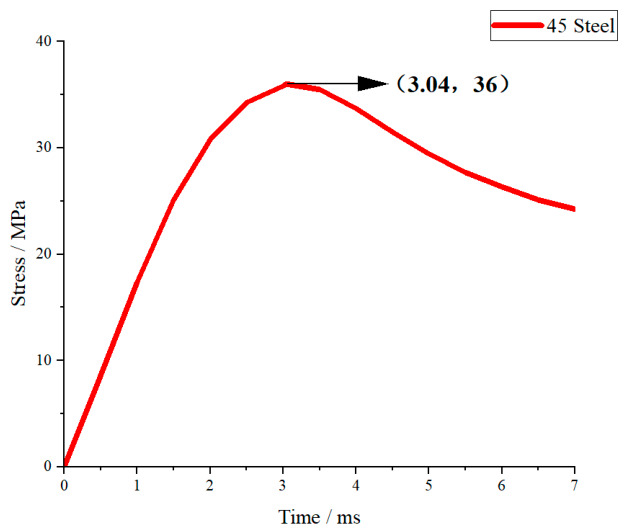
45 Steel stress–time curve.

Under the same impact condition with a drop height of 1.5 m (corresponding to a peak impact force of approximately 5000 N), the transient stress–time curves of fiberglass (EP), aluminum alloy (6061-T6), structural steel (Q420), and 45 steel were obtained by numerical simulation, as shown in Figure 7, Figure 8, Figure 9 and Figure 10.

#### 3.2.1. Single Stress Response Analysis of Each Material

As shown in Figure 7, the stress–time curve of fiberglass (EP) rises rapidly and reaches the peak stress at about 3 ms. The peak stress is the highest among the four materials, close to the tensile strength of the material. After the peak, the stress drops quickly and the attenuation rate is high. It is worth noting that although fiberglass exhibits a high peak stress, its maximum impact force is significantly lower than that of metallic materials. This is mainly because the elastic modulus of fiberglass is much lower than that of steel and aluminum alloy. A low elastic modulus greatly reduces the equivalent structural stiffness, resulting in greater deformation and longer action duration during impact. The impact force is effectively buffered and dispersed, thereby markedly reducing the peak impact load. The stress response is strong but the stability is poor, which is consistent with the brittle failure characteristics under impact.

As shown in Figure 8, the stress curve of 6061-T6 aluminum alloy rises gently and reaches the peak at about 3 ms. The peak stress is moderate, slightly higher than the yield strength of the material, showing a small range of elastic–plastic response. After the peak, the stress decreases slowly and the attenuation process is smooth. Aluminum alloy has moderate stiffness and good plasticity; so, the stress growth is uniform and the impact response is stable, which reflects the good deformation coordination ability of metallic materials.

As shown in Figure 9, the stress curve of Q420 structural steel rises slowly and the peak stress is the lowest among the four materials. The peak appears at about 3 ms and is far below the yield strength, maintaining in the elastic stage all the time. After the peak, the stress decreases gently and the curve is stable with small fluctuations. Q420 has high elastic modulus and high yield strength; so, the structure stiffness is large, the deformation is small under the same impact, and the stress level is significantly suppressed.

As shown in Figure 10, the stress–time curve of 45 steel is similar to that of Q420. The stress rises steadily and reaches the peak at about 3 ms. The peak value is slightly higher than Q420 but still at a low level and remains in the elastic stage. The post-peak attenuation is gentle and the curve fluctuation is small. Since 45 steel has the same elastic modulus as Q420 and similar strength level, its stress response law is close to Q420, with good stability and low stress amplitude.

#### 3.2.2. Overall Comparative Analysis of Stress Responses

Under the same impact load, the stress responses of the four materials show obvious differences determined by elastic modulus, yield strength and plasticity. In terms of peak stress, the order from high to low is: fiberglass (EP) > aluminum alloy (6061-T6) > 45 steel > structural steel (Q420). In terms of response stability, the order from good to poor is: Q420 structural steel > 45 steel > 6061-T6 aluminum alloy > fiberglass (EP).

Materials with a higher elastic modulus (steel) have greater structural stiffness, smaller deformation under impact, and lower peak stress and better stability. Aluminum alloy has moderate stiffness and takes into account lightweight and response stability. Fiberglass has the lowest stiffness, leading to the highest stress amplitude and rapid attenuation, showing obvious brittle material characteristics. The above stress response laws are consistent with the theoretical analysis of energy absorption and impact force, and can provide a direct mechanical basis for material selection of crossover frame structures.

### 3.3. Analysis of the Energy Absorption Effect of the Main Material

**Figure 11:**
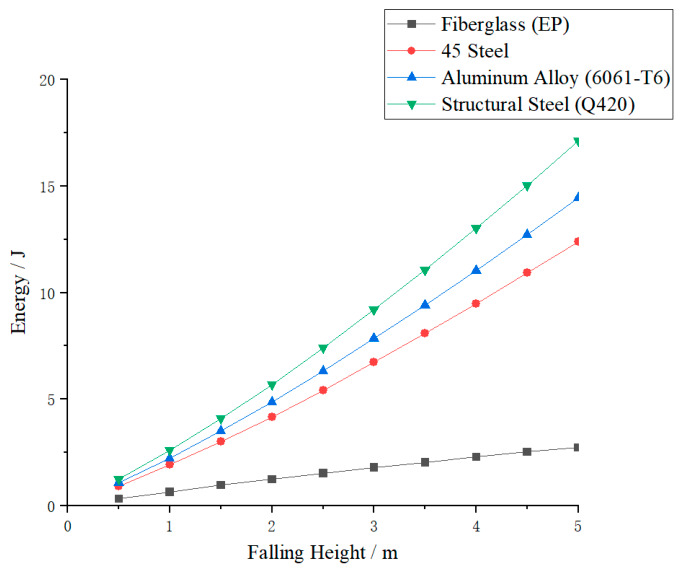
Energy variation curve of crossover frame crossbeam with falling height.

**Figure 12:**
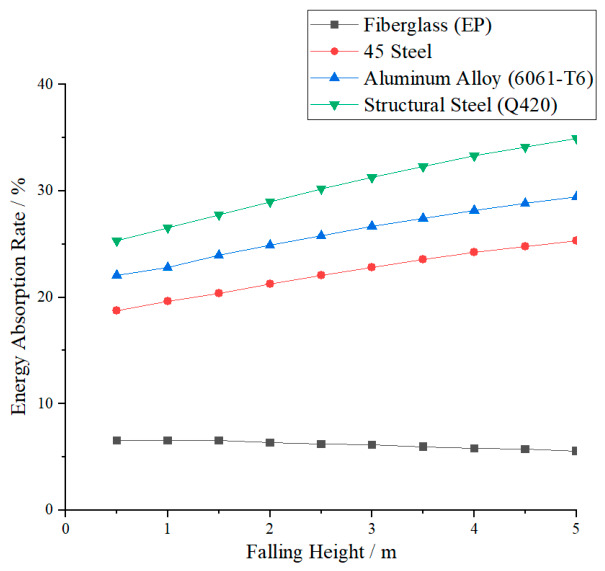
Comparison of energy absorption rates of crossover frame crossbeams made of different materials with changes in fall height.

Under the undamped setting, the experiment collected energy absorption data of four types of material crossover frames under impact loads. The relationship between energy absorption and drop height is shown in Figure 11, and the comparison of energy absorption rates under different drop heights is presented in Figure 12. As can be seen from the figures, the energy absorption of the crossover frames for all materials continues to increase with the rise in drop height, reflecting a positive correlation between impact kinetic energy and structural energy absorption. Further comparison shows that there is a significant difference in energy absorption levels among different materials, and the energy absorption capacity ranks from high to low as follows: structural steel Q420, aluminum alloy 6061-T6, 45 steel, and fiberglass EP. This difference indicates that the mechanical properties of the materials themselves have a significant impact on impact response, and materials with high strength and sufficient plastic deformation can achieve more stable and efficient energy absorption.

In terms of the overall energy absorption characteristics of the four materials, the energy absorption and mechanical response characteristics of the four material crossover frame crossbeams under undamped impact conditions show significant differences. Structural steel Q420, with the highest yield strength (420 MPa) and the longest plastic strain range (elongation 18%), has the strongest energy absorption capacity among the four materials. At a 5 m drop height, its absorbed energy can reach a high proportion of the impact kinetic energy, making it suitable for heavy-duty scenarios requiring high total energy dissipation. However, its high modulus also results in a relatively large peak impact force. Aluminum alloy 6061-T6 ranks second in energy absorption capability, with excellent specific energy absorption (energy absorption per unit mass), uniform and controllable deformation, and stable energy absorption efficiency, making it suitable for applications with lightweight requirements. Although its total energy absorption is lower than that of Q420 steel, its lightweight advantage makes it a preferred choice for weight-sensitive designs. 45 steel ranks third in energy absorption capability; its post-yield hardening characteristics contribute to plastic energy absorption, giving it acceptable overall performance for conventional working conditions. Glass-fiber-reinforced plastic (EP), as a brittle material (elongation only 2.0%), has the weakest material energy absorption. It mainly relies on elastic deformation and cannot dissipate energy through plastic mechanisms. However, in impact response, its low elastic modulus (22 GPa) significantly reduces the structural equivalent stiffness, thereby effectively lowering the peak tension—this is a structural stiffness effect rather than material energy absorption. Peak tension reduction is achieved at the cost of large structural deformation, and exceeding the elastic limit may lead to brittle fracture; thus, careful evaluation is required for primary structural applications.

To further quantify the energy absorption and mechanical response characteristics of crossings made of different materials, this study calculates the percentage of energy absorbed by the crossing relative to the impact kinetic energy (energy absorption efficiency) under various working conditions, and plots the energy absorption comparison curves at different drop heights, as shown in Figure 11. Analysis shows that the energy absorption efficiency of the four materials is consistent with the energy absorption capacity ranking: structural steel Q420 > aluminum alloy 6061-T6 > 45 steel > fiberglass EP. Among them, Q420 structural steel has the most significant energy absorption effect, and its energy absorption efficiency increases steadily with the increase in drop height. The energy absorption efficiency of 6061-T6 aluminum alloy is lower than that of Q420 steel but higher than that of 45 steel, with a smooth and stable change trend. 45 steel takes the third place with moderate energy absorption efficiency. Fiberglass EP has the lowest energy absorption efficiency, which even decreases slightly with increasing drop height. In addition, as drop height increases, the energy absorption efficiency of the three metallic materials generally increases, indicating that larger impact energy can more fully activate the plastic dissipation mechanisms, and the performance gap between different materials further widens accordingly.

In summary, the selection of materials should clarify the design objectives: if the primary goal is total energy dissipation, structural steel Q420 is optimal; if the goal is lightweight design and stable energy absorption, aluminum alloy 6061-T6 has the best overall performance; 45 steel can be used in conventional non-critical structures; although fiberglass can significantly reduce peak force, its energy absorption capacity is limited and it carries a risk of brittle fracture, making it unsuitable for the main structure of a nondamped spanning frame with high reliability requirements.

## 4. Simulation Analysis

### Simulation Verification

**Figure 13:**
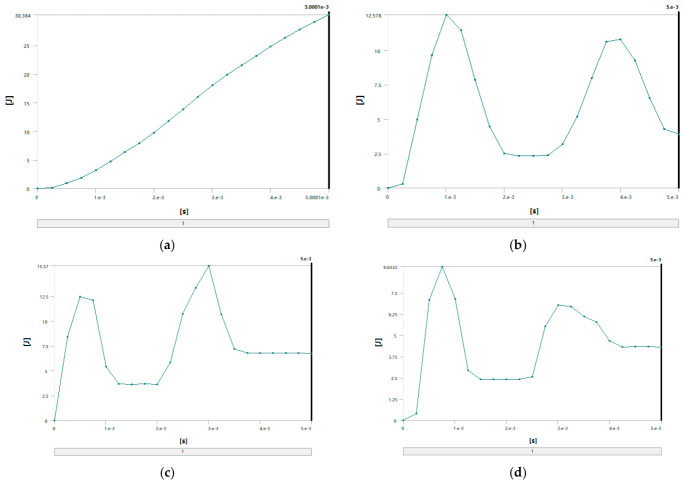
Energy simulation of four materials: (**a**) fiberglass; (**b**) aluminum alloy; (**c**) structural steel; (**d**) 45 steel.

**Figure 14:**
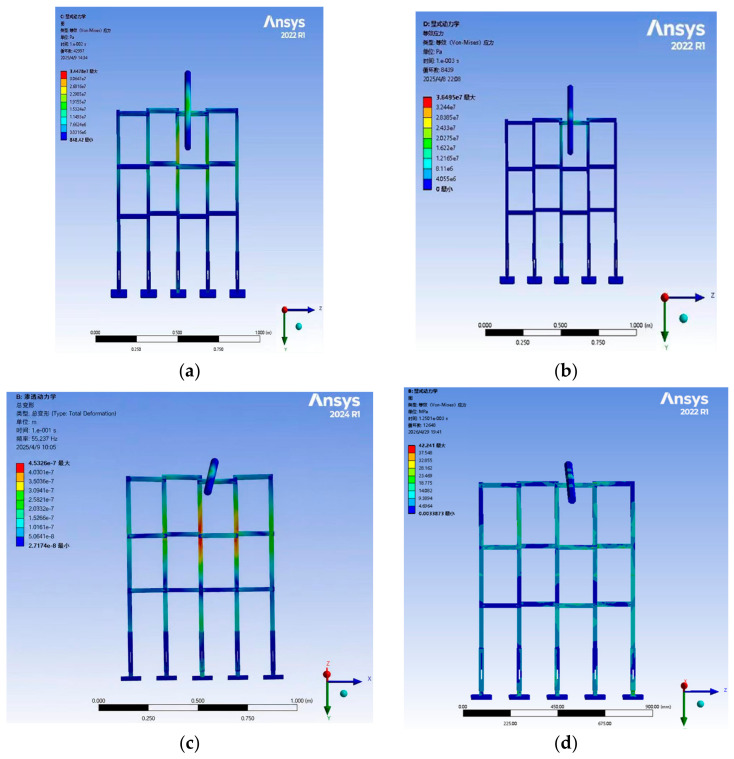
Stress nephograms of four types of materials: (**a**) structural steel (**b**) aluminum alloy; (**c**) 45 steel; (**d**) fiberglass. The Chinese text in the upper left corner of the figure represents the following meanings from top to bottom: B or C or D: explicit dynamic; equivalent stress (Since different versions of ANSYS are used, BCD appears, but their meanings are the same); type: equivalent stress; units; time; cycle number; Max; Min.

To verify the above conclusions, ANSYS simulation software was employed to further analyze the energy absorption effects and stress of different materials. With a falling height set at 1.5 m and a collision time of 0.005 s, the energy absorption and stress of four materials crossover frame are shown in Figure 13 and Figure 14. The theoretical collision time is 0.001 s. From Figure 13 and Figure 14, the following results can be obtained: when using fiberglass (epoxy resin EP), the energy absorption of the crossover frames is 3.6 J and the stress magnitude is 45.72 MPa; if aluminum alloy (6061-T6) is used, the energy absorption is 12.578 J and the stress magnitude is 36.49 MPa; if structural steel (Q420) is selected, the energy absorption is 13.012 J and the stress magnitude is 34.48 MPa; and when using 45 steel, the energy absorption is 9.0332 J and the stress magnitude is 41.68 MPa.

Based on the above simulation data, the energy absorption effects and stress of crossover frame crossbeams with different materials can be further compared and analyzed. Under the same impact condition with a falling height of 1.5 m, the energy absorption values and stress of the crossbeams made of the four materials are as follows: fiberglass (EP), 3.6 J, 45.72 MPa; aluminum alloy (6061-T6), 12.578 J, 36.49 MPa; structural steel (Q420), 13.012 J, 34.48 MPa; and 45 steel 9.0332 J, 41.68 MPa. The simulation results show that the energy absorption capacity in descending order is: Q420 structural steel > aluminum alloy > 45 steel > fiberglass. This sequence is highly consistent with the intrinsic mechanical properties of the materials: Q420 structural steel has the highest yield strength and the largest plastic elongation, enabling efficient energy dissipation through sufficient plastic deformation with optimal energy absorption capacity; aluminum alloy features uniform deformation and outstanding specific energy absorption, presenting excellent overall energy absorption; 45 steel exhibits stable elastoplastic energy dissipation capacity, with slightly lower energy absorption than Q420 structural steel; fiberglass is a brittle material that dissipates energy mainly through elastic deformation without a plastic energy dissipation mechanism, resulting in the weakest overall energy absorption capacity. The above simulation results can provide quantitative support for material selection of the main structure of undamped crossover frames: Q420 structural steel is preferred when impact energy dissipation is the core objective; aluminum alloy shows the best comprehensive performance in scenarios requiring a balance between lightweight design and energy absorption; fiberglass has limited energy absorption capacity but can significantly reduce peak tension due to its low modulus, making it suitable for occasions with specific load control requirements.

## 5. Conclusions

This paper takes the undamped crossover frame as the primary research object, and selects four commonly used engineering materials—fiberglass (epoxy resin, EP), aluminum alloy (6061-T6), structural steel (Q420), and 45 steel—as the main material variables. The ultimate experimental parameters were determined through multiple rounds of theoretical calculations and numerical simulation iterations. A 1:20 scaled model was fabricated and tested under conductor impact loads to investigate the impact response characteristics of crossover frames with different materials, as well as the influence of drop height on their energy absorption and mechanical responses. Quantitative analysis was performed on the performance differences among the four materials so as to provide experimental support and data basis for material selection of the main body of undamped crossover frames. The main conclusions are as follows:

(1) With the structural configuration of the crossover frame unchanged, the four materials exhibit significant differences in energy absorption and mechanical response characteristics, which should be evaluated from two dimensions:
  ① In terms of peak tension reduction, with Q420 structural steel as the reference, fiberglass (EP) crossover frame shows the most remarkable tension reduction effect, owing to its low elastic modulus (22 GPa) that reduces the equivalent structural stiffness and thus effectively mitigates impact loads. Nevertheless, affected by the anisotropic nature of the material, its response stability fluctuates slightly under certain drop heights. The tension reduction in aluminum alloy (6061-T6) crossover frame is slightly lower than that of fiberglass, but it presents better overall stability and maintains a steady response within a wider range of impact energy. The tension reduction in Q420 structural steel is close to that of 45 steel; with the same elastic modulus and similar strength grade, the two steels demonstrate analogous stress response laws and favorable stability.
  ② In terms of energy dissipation (energy absorption efficiency), the order is opposite to that of tension reduction: Q420 structural steel possesses the strongest energy absorption capacity, with an efficiency of approximately 35%, which is attributed to its highest yield strength (420 MPa) and the longest plastic deformation range, enabling sufficient dissipation of impact energy through plastic deformation. Aluminum alloy ranks second with an energy absorption efficiency of about 26%, and features excellent specific energy absorption (energy absorption per unit mass), making it suitable for lightweight-demanding scenarios. 45 steel has an energy absorption efficiency of around 22%. As a typical brittle material (elongation only 2.0%), fiberglass (EP) shows the weakest energy absorption capacity, with an efficiency of merely 6%, since it mainly relies on elastic deformation and cannot dissipate energy via plastic mechanisms.

The above results reveal that peak tension control and energy dissipation are two distinct performance objectives: fiberglass excels in load reduction, while steel performs best in energy absorption. A trade-off selection should be made according to specific design requirements in engineering applications.

(2) As the conductor drop height increases (0.5–5.0 m), the tensile force of the four types of crossover frames rises continuously. For metallic materials (structural steel, 45 steel, and aluminum alloy), the energy absorption efficiency increases with the drop height, indicating that larger impact energy can more sufficiently activate the plastic energy dissipation mechanisms of metals, and the gap in energy absorption performance among different materials is further widened. In contrast, the energy absorption efficiency of fiberglass decreases slightly with increasing height, reflecting the inherent characteristics of brittle materials: once exceeding the elastic limit, it is prone to damage and fails to sustain energy absorption [22].
(3) With the Q420 structural steel crossover frame as the benchmark, the maximum drop height for conductor impact is determined to be approximately 5 m via simulation. The test drop height is set in the range of 0.5–5.0 m with a 0.5 m increment for step-by-step loading. It is verified that within this height range, the crossover frames made of the four materials all possess sufficient strength to bear the impact tension of the conductor without exceeding their ultimate bearing capacity. This can provide a reference for parameter setting in subsequent impact tests of damped crossover frames.
(4) It should be emphasized that the 1:20 scaled model tests conducted in this study are inherently a comparative investigation across different materials. The reported quantitative values of impact force and absorbed energy are valid for ranking the relative performance of the four materials and for revealing the underlying trends, but they cannot be directly extrapolated to full-scale engineering design through simple proportionality. Severe gravity distortion and potential strain-rate effects [23] are intrinsic limitations of reduced-scale impact testing under normal gravity. Future work should aim at conducting selective full-scale destructive tests for validation or developing multi-scale finite element simulations that incorporate rate-dependent material constitutive models so as to establish a more reliable cross-scale prediction framework.

In the design and application of crossarms for future power engineering projects, rational selection of primary materials is critical to achieving design targets. Fiberglass and aluminum alloy are advantageous if the primary goal is to control crossarm tension and mitigate impact loads. Steel (especially Q420) is optimal if the core objective is to dissipate impact energy and ensure structural safety. Aluminum alloy is the preferred choice when both lightweight and energy absorption are required. The research findings on the energy absorption and mechanical response characteristics of crossover frames with different materials can offer experimental support and theoretical foundation for the selection of main materials for damped crossover frames.

## Figures and Tables

**Table 1:** Crossover frame system parameters.

Parameter	Value or Material
Spacing between vertical members/mm	170
Single-sided width	680
Spacing of horizontal members/mm	250
Total frame height/mm	1000
Insulating protective net	UHMWPE rope
Insulating mat lateral dimension/mm	680
Roof-supporting crossbeam	Nylon rod
Frame material	Fiberglass (EP), aluminum alloy (6061-T6), structural steel (Q420), 45 steel
Transmission Line Specifications	JL1/G1A-630/45
Conductor Span/m	1

**Table 2:** Material parameters.

Parameters	Fiberglass	Aluminum Alloy	Structural Steel	45 Steel
Density/g·cm^−3^	18	2.7	7.85	7.85
Elastic Modulus/GPa	22	68.9	210	210
Tensile Strength/Mpa	350	310	600	600
Yield Strength/Mpa		270	420	355
Elongation/%	2	10	18	16

**Table 3:** Required vs. actual scale factors for the 1:20 impact test.

Physical Quantity	Required Similarity Factor (Elastic–Inertial)	Actual Factor in Experiment
Length, L	1/20	1/20
Elastic Modulus, E	1	1
Mass, M	1/500	≈1/500
Stiffness, k	1/20	≈1/20
Time, t	1/20	Not independently controlled
Velocity, v	1	$1/\sqrt{20}\approx 0.224$
Drop Height, h	1	1/20
Stress, σ	1	—
Strain Rate, $\;\dot{\varepsilon }$	20	≈4.47

## Data Availability

The original contributions presented in this study are included in this article. Further inquiries can be directed to the corresponding author.

## Abbreviations

E_k_	Conductor Kinetic Energy
m	Mass of the Wire
g	Acceleration Due to Gravity
U	Deformation Energy of the Crossbar
δ	Vertical Displacement of the Top Crossbar of the Scaffolding
T_max_	Maximum Tension of the Top Crossbar of the Truss
k	Equivalent Stiffness
E	Elastic Modulus
ω_e_	Elastic Strain Energy Density
σ_y_	Yield Strength
ω_p_	Plastic Dissipation Energy
ε_y_	Yield Strain
ε_u_	Ultimate Strain
σ(ε)	Flow Stress
η	Energy Absorption Efficiency
E_1_	The Energy of the Crossbar
E_2_	The Energy of the Wire
